# Characterization and Comparison of Extra Virgin Olive Oils of Turkish Olive Cultivars

**DOI:** 10.3390/molecules28031483

**Published:** 2023-02-03

**Authors:** Aziz Korkmaz

**Affiliations:** Department of Nutrition and Dietetics, Faculty of Health Sciences, Mardin Artuklu University, Mardin 47200, Turkey; azizkorkmaz@artuklu.edu.tr

**Keywords:** olive oil, phenolic compound, volatile compound, PCA, lipoxygenase, fatty acid, chlorophyll, carotenoid, antioxidant capacity, olive cultivar

## Abstract

Extra virgin olive oils (EVOOs) obtained from five Turkish olive cultivars widely produced in the Aegean and Marmara regions were investigated based on their total antioxidant capacity (TAC), total phenolic content (TPC), pigment contents, fatty acid (FA) profiles, phenolic compounds (PC), volatile compounds (VC), and sensory properties. The results showed that all properties of EVOO samples were significantly affected by the olive cultivar used. The pigment contents in Ayvalık (9.90 mg·kg^−1^) and Uslu (9.00 mg·kg^−1^) oils were higher than the others (*p* < 0.05). The greatest values for oleic acid (74.13%) and TPC (350.6 mg·kg^−1^) were observed in Gemlik and Domat oils, respectively (*p* < 0.05). Edincik oil showed the maximum hydroxytyrosol content (48.022 mg·kg^−1^) and TAC value (515.36 mg TE·kg^−1^) (*p* < 0.05). The Edincik, Domat, and Uslu oils were significantly not different for the total content of C6 compounds derived by lipoxygenase, which are the main volatiles responsible for the typical aroma of EVOOs (*p* > 0.05). Domat oil also exhibited the highest scores for bitterness and pungency perceptions (*p* < 0.05). The fruitiness scores of the oil samples (except for Ayvalık oil) were close to each other, even if they were statistically different (*p* < 0.05). Principal component analysis (PCA) indicated that the Ayvalık oil was separated from the others due to its poor-quality characteristics. As a result, it can be stated that Domat olive oil has better quality than the others.

## 1. Introduction

Extra virgin olive oil (EVOO) is obtained only via mechanical or physical processes from the olive fruit (*Olea europaea* L.) and is designated as the highest quality in the classification of olive oils (OO) [1]. The main popularity of EVOO comes from its pleasant flavor and various beneficial effects on health [2]. In particular, advances in healthy diets have increased the demand for olive oil globally from year to year.

Nowadays, some comprehensive components, especially the profiles of volatile, phenolic, and pigment constituents, are used in the quality assessment of EVOO. In recent years, these components have been widely used to determine the characterizations [3,4] and authentications [5,6] of monovarietal EVOOs. Volatile compounds (VC) in EVOO are mainly responsible for its typical flavor [4], while PCs are associated with their pharmacological effects on health [7]. VCs are mostly formed by lipoxygenase activity during the initial stage of EVOO extraction [8].

The positive effects of EVOO on health are mainly attributed to its fatty acid (FA) fractions and PC [8]. It is reported that PC in EVOO can both prevent the oxidation of low-density lipoprotein (LDL) and reduce its amount in blood plasma [8]. Therefore, the European Commission (EC, 2012) [9] allowed the health claim on the proposition of the European Food Safety Authority (EFSA) [10] that offers the consumption of an amount containing at least 5 mg of hydroxytyrosol and its derivatives per 20 g of EVOO. These molecules also play crucial roles in the gustatory perception and shelf life of EVOO [2]. Moreover, phenolic compounds contribute to prolonging the shelf life of the EVOO by increasing its oxidative stability [11].

Turkey is one of the countries where olive farming has been carried out for a long time. According to a hypothesis that is generally accepted, the origin of the wild olive tree is a certain region of Turkey (as known Anatolia or Minor Asya), and olive cultivation spread from Syria to Greece and other Mediterranean countries through this area, which includes the Aegean region [12,13]. Additionally, archaeological digs revealed an ancient OO mill dating from 600 BC in Urla, a county of Izmir Province located in this region [12]. In 2021, Turkey was the fourth-largest country in the world in terms of total OO production [14]. Almost half (48%) of its total annual olive production is obtained in the Aegean region [15].

Numerous comparative studies have been conducted on the main properties of different monovarietal EVOO. However, many of them have investigated only a certain class of components or routine quality characteristics of EVOO. Diraman et al. (2010) [16] studied the relationship between FA compositions and geographical origins of 103 Turkish OO samples. In another research, the influences of cultivar, harvest year, and geographical location on FA fractions in a total of 36 VOO samples produced from 18 various olive cultivars from different countries were also investigated by Hmida et al. (2022) [17]. Dogi et al. (2020) determined PC in virgin olive oil (VOO) of six different Albanian olive cultivars [18]. PC and VC in different Italian cultivars of EVOO were determined by Veneziani et al. (2018) [8]. Ilyasoglu et al. (2010) characterized the EVOO of two prominent cultivars of the Aegean region of Turkey based on their VC [19]. Moreover, Kaftan and Elmaci (2011) compared a total of 30 VOO from 2 varieties in terms of VC and legal quality parameters [20].

In the literature, studies on the comprehensive characterization and comparison of monovarietal Turkish EVOO are limited. Although there have been various studies on some features of the country EVOO, these mostly focused on only one of their component groups, such as PC, VC, and FA fractions. In this sense, this study aimed to determine and compare the VC, PC, and FA profiles, TAC, pigment contents, and sensory properties of the EVOO of Turkish olive cultivars widely grown in the Aegean and Marmara regions.

## 2. Results and Discussion

### 2.1. Quality Parameters

FFA, PV, K_232_, and K_270_ are the basic parameters used in the classification of olive oils. Among these, FFA is a measure of the enzymatic hydrolysis of lipids, while the others are indicators for determining the level of oxidation in lipids [21]. These features are mainly affected by the maturity degree of olives and extraction conditions used in the production of oils, as well as by the variety of olive [22,23]. The FFA, PV, K_232_, and K_270_ of the oils ranged from 0.05 ± 0.00 to 0.12 ± 0.01%, 3.50 ± 0.27 to 6.33 ± 0.72 meq O_2_·kg^−1^, 1.64 ± 0.01 to 1.99 ± 0.02, and 0.13 ± 0.00 to 0.18 ± 0.02, respectively (Table 1). Based on these ranges, all oil samples were classified as EVOO according to European Commission (EC,2003) [24] and International Olive Council (IOC, 2021) [25]. Additionally, the values observed for the quality parameters of the samples were in agreement with the previous findings on most of the EVOOs of both Turkish [26,27] and other countries [28,29].

### 2.2. Pigment Contents

The concentration of chlorophyll and carotenoid in OOs directly affects their color [30]. The OOs with a higher intensity of green color are generally preferred by consumers, as this color is associated with freshness and good quality [23]. The amount of chlorophyll in oils ranged from 1.84 ± 0.12 to 5.52 ± 0.01 mg·kg^−1^, while the carotenoid content varied from 1.67 ± 0.05 to 4.47 ± 0.02 mg·kg^−1^ (Table 1). Both chlorophylls and carotenoids contents of Ayvalık and Uslu oils were higher than those of other variety olive oils, while lower these contents were found in Domat and Gemlik oils (*p* < 0.05).

The amount of these pigments in OOs can vary widely depending on the primarily variety and harvest time [22] and also climatic conditions and processing methods [31]. Jolayemi et al. (2021) [32] reported that chlorophyll and carotenoid contents of a hundred samples of Turkish OOs from different olive cultivars obtained from five different production seasons varied from 0.6 to 5.6 and 0.6 to 3.3 mg·kg^−1^, respectively, as a wide range. The pigment contents in the current study also showed wide ranges. In a previous study by Jolayemi et al. (2016) [22], it was reported that ranges of chlorophylls and carotenoids in Memecik oils were 1.50–4.55 and 1.11–2.86 mg·kg^−1^ and in Ayvalık oils were 1.28–2.57 mg·kg^−1^ and 1.01–1.61 mg·kg^−1^, respectively. These values reported for Ayvalık oil are lower than those found for the same variety in the present study.

### 2.3. Fatty Acid Profile

Fatty acid compositions of the EVOO samples are given in Table 2. The amounts of fatty acid fractions in all samples fulfilled the legal ranges for EVOO stated in EC (2003) [24] and IOC (2021) [25]. The amounts of oleic acid in all samples were the highest, followed by palmitic, linoleic, and stearic acids. There were significant differences between these major fatty acid levels in the oil samples (*p* < 0.05). A rich oleic acid content in oils is not only desirable for a long shelf life concerning oxidative stability but is also necessary for health benefits [26]. In fact, the oleic/linoleic acid ratio can be used as an index to measure the stability of olive oil, with a high ratio indicating good oxidative stability for oils [33]. The value of this ratio for Gemlik oil (13.65) was clearly higher than that of the others, which ranged between 6.11(Uslu) and 7.41 (Ayvalık). Because of its higher oleic acid content, Gemlik oil also exhibited the highest MUFA (75.61 ± 0.03%), lowest PUFA content (6.10 ± 0.02) and thus showed a higher MUFA/PUFA ratio (12.4 ± 0.03). Palmitic acid was the main unsaturated fatty acid in all oils, with a range of 12.88 ± 0.01% (Edincik) to 14.58 ± 0.01% (Ayvalık).

The composition of fatty acids in OOs is mainly affected by genetic variations, geographical conditions (locations, climate, latitude), ripening degree, season, and production conditions [32,34]. The amount of oleic acid in the Ayvalık oil of this study was in agreement with the results of Uluata et al. (2021) [26], who found a range between 52.78 ± 2.03% and 71.22 ± 1.12% for oleic acid levels in different eleven commercial EVOOs from the Ayvalık olive cultivar grown in the Aegean region. Jolayemi et al. (2021) [32] reported that MUFA, PUFA, and MUFA/PUFA values in a hundred OO samples obtained from 8 different olive cultivars (including Ayvalık) from various locations in Aegan region ranged from 66 to 76.5%, from 8.6 to 18.2%, and from 3.6 to 8.8, respectively, in agreement with the findings of this study. Another study on the relationship between the origin, harvest year, and fatty acid profile of Turkish VOOs (Diraman et al., 2010) [16] reported ranges for oleic, palmitic, linoleic, stearic, and linolenic acid percentages as 62.90–77.16, 9.62–18.97, 6.26–17.17, 1.42–3.54, and 0.37–1.00, respectively, in 103 VOOs from numerous olive cultivars, including the Ayvalık, Gemlik, and Domat varieties. These findings are consistent with those in this study. Similar results regarding fatty acid fractions have also been reported for EVOOs from different countries [33,35]. Consequently, the differences between fatty acid fractions in the EVOO samples may not only be due to the variety effect but also to other factors mentioned above.

### 2.4. Individual Phenolic Compounds and Total Phenolic Content (TPC)

The PCs in EVOO are phenolic alcohols (hydroxytyrosol, tyrosol) and their secoiridoid derivatives, phenolic acids, flavonoids, and lignans [12]. Among these, hydroxytyrosol, tyrosol, and their secoiridoid derivatives, and the lignans are responsible for the pungency and bitterness of EVOO [9,13]. The individual PC contents and TPC in EVOO samples are presented in Table 3. EVOO from Domat contained the highest TPC (350.6 ± 8.770 mg·kg^−1^), followed by Gemlik (321.905 ± 6.185 mg·kg^−1^), Edincik (294.090 ± 13.810 mg·kg^−1^), Uslu (291.145 ± 5.485 mg·kg^−1^), and Ayvalık (234.71 ± 1.490 mg·kg^−1^) olives. These values were higher than the TPC levels reported for five different EVOO obtained from the Chemlali variety (117.64 ± 1.23–151.7 ± 1.97 mg GAE·kg^−1^) grown in different locations [36]. TPC in the Ayvalık oils was close to that found by Uluata et al. (2021) [26], who reported that the TPC in EVOO from Ayvalık olives grown in a location close to that used in this study was 215.29 ± 1.55 mg GAE·kg^−1^. However, the TPC determined by these authors for EVOO from Uslu (114.22 ± 3.44 mg GAE·mg^−1^) was lower than that of this study. These differences could mostly be due to the variety [37], maturity [22], and growing conditions of olives [21].

A total of 11 phenolic compounds were identified and quantified in the oil samples (Table 3). The concentrations of most of them were significantly affected by cultivars (*p* < 0.05). Many previous studies have also reported that the cultivar affects the phenolic compound content of OOs [27,29]. Phenolic acids were scarce in all oils in comparison with other phenolic groups, as found by previous studies [23,38]. Among the phenolic acids identified in samples, vanillic acid was the most abundant compound, with a range of 1.445 ± 0.220 (Uslu) to 1.991 ± 0.031 mg·kg^−1^ (Edincik), while caffeic acid had the lowest content (0.009 ± 0.001 to 0.184 ± 0.002 mg·kg^−1^). The phenolic acid contents determined in all samples are in agreement with those reported in many previous studies [35,39].

Hydroxytyrosol was the predominant PC in the samples, with an amount in the range of 11.621 ± 0.420 to 48.022 ± 1.186 mg·kg^−1^, except in Ayvalık oil, which contained the greatest amount for pinoresinol (15.247 ± 0.524 mg·kg^−1^) within the PC. The amount of hydroxytyrosol in Edincik oil (48.022 ± 1.186 mg·kg^−1^) was significantly higher than that in other oils (*p* < 0.05). The level of hydroxtyrosol in the EVOO from Ayvalık olive (11.621 ± 0.420 mg·kg^−1^) was greater than that reported by Jolayemi et al. (2016) [22] for nine EVOO samples obtained by three different malaxation temperatures (27, 37, and 47 °C) from Ayvalık cultivar harvested at three different times. Additionally, the amounts of hydroxytyrosol in their EVOO samples were lower than those of tyrosol, contradicting that of the analyzed oils. The tyrosol contents in all samples were similar to that in the EVOOs of six different Italian olive varieties reported in a previous study [8], whereas the hydroxtyrosol contents observed were higher than theirs. The hydroxytyrosol content in OOs of Moroccan, Spanish, and Tunisian cultivars was reported as 6.65, 14.34, and 22.57 mg GAE.kg^−1^, respectively, by Negro et al. (2019) [40]. On the other hand, Topi et al. (2020) [18] reported that the hydroxytyrosol contents in five Albanian OO samples were higher than that of tyrosol, similar to the findings of the present study. As is known, hydroxytyrosol and tryrosol are derived by enzymatic hydrolysis from oleuropein and ligstroside, respectively [41]. Additionally, it was reported that the antioxidant activity of hydroxytyrosol is better than that of tyrosol [42,43]. In fact, there was a high correlation between the hydroxytyrosol content and the TAC of the samples (Pearson’s coefficient = 0.979, *p* < 0.001), but tyrosol was not interestingly correlated with TAC (*p* > 0.05).

Regarding flavonoids, EVOOs from Domat and Gemlik olives provided higher apigenin contents compared to the other cultivars. Apigenin and luteolin concentrations in the oils ranged from 2.409 ± 0.030 (Edincik) to 12.824 ± 0.010 mg·kg^−1^ (Gemlik) and from 1.640 ± 0.089 (Edincik) to 2.658 ± 0.080 mg·kg^−1^ (Ayvalık), respectively. The apigenin contents of the five EVOOs were more than that of two fresh monovarietal EVOOs from Portuguese olive varieties (0.71–1.00 mg·kg^−1^) reported by Klisović et al. (2022) [44], but the luteolin contents were lower than theirs (3.05–3.73 mg·kg^−1^). Moreover, the value of luteolin found in the EVOO from Ayvalık (2.658 ± 0.080 mg·kg^−1^) was partially less than the range (3.65–5.24 mg·kg^−1^) reported by Jolayemi et al. (2016) [22] for EVOOs of the same variety produced under similar conditions concerning malaxation temperatures and maturity. The amounts of PC in OOs are affected by genetic as well as pre- and post-harvest factors [45]. In a previous study [27], the amount of apigenin in Ayvalık and Gemlik oils varied from 4.74 ± 0.49 to 6.71 ± 0.73 mg·kg^−1^ and from 1.00 ± 0.04 to 1.65 ± 0.11 mg·kg^−1^, respectively. These contents are lower than those of the oils from same cultivars investigated in the current study.

As a noticeable point, although the amounts of major secoiridoids (hydroxytyrosol and tyrosol derivatives) in the samples based on their HPLC peaks have not been quantified, it is estimated that most of the TPC measured by spectrophotometry consisted of amounts of these compounds [42]. Essentially, in many previous studies on OOs, TPC values calculated spectrophotometrically were lower than the sum of individual PCs identified by HPLC [29,46,47]. Because of this, it can be concluded that the EVOOs from the olive varieties (except for Ayvalık) fulfilled the value of PC required by the European Commission for the health claim [9], which requires at least 5 mg of hydroxytyrosol and its other secoiridoid derivatives in 20 g of OO or at least 250 mg in 1 kg of OO for these PC as an equivalent concentration.

### 2.5. Total Antioxidant Capacity (TAC)

**Figure 1:**
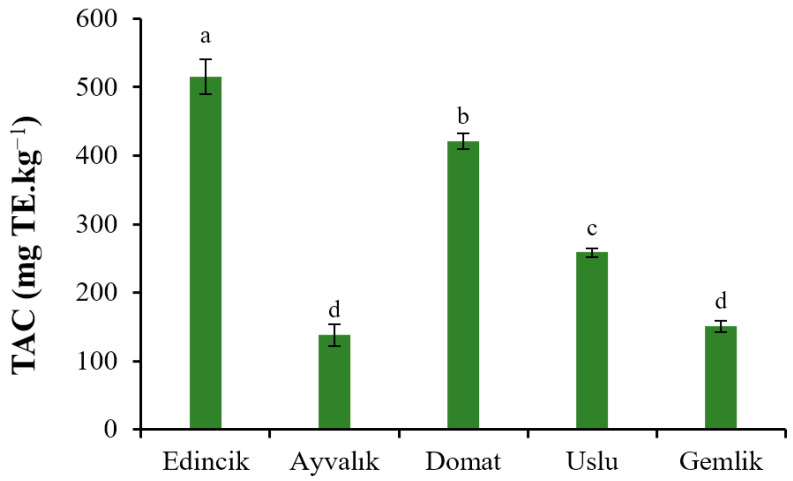
TAC of EVOOs from five Turkish olive cultivars. Different letters on the bars indicate significant differences between different oil samples (*p* < 0.05).

Certain PCs in OOs, such as oleuropein derivatives, exhibit strong antioxidant activities based on their radical scavenging abilities, as well as some other useful biological functions. The antioxidant effects of these PCs also contribute to the extended shelf life of OOs against lipid oxidation in comparison with those of other vegetable oils [48]. The TAC of the sample in terms of 2,2-Diphenyl-1-picrylhydrazyl (DPPH) scavenging capacity are shown in Figure 1. The TAC of the Edincik, Domat, Uslu, Gemlik, and Ayvalık oils were 515.36 ± 25.62, 420.98 ± 11.36, 258.63 ± 6.37, 150.66 ± 8.49, and 137.91 ± 16.23 mg TE·kg^−1^. It can be seen that the TAC of Edincik and Domat oils were notably higher than those of the other three oils.

In a recent study, Comlekcioglu et al. (2022) [37] reported that differences between the DPPH scavenging capacity of Gemlik (80 ± 3.6%), Ayvalık (75.92 ± 4.2%) and Domat olives (74.00 ± 3.3%) were not significant (*p* > 0.05), agreeing with the case between the EVOOs of Ayvalık and Gemklik olives. The TAC of Edincik and Ayvalık oils was higher than that reported by Uluata et al. (2021) [26], who found that the TACs (DPPH method) for ten different EVOOs of Ayvalık variety harvested from various locations of the Aegean region were between 58.25 ± 0.00 and 309.03 ± 14.02 mg TE·kg^−1^. They also reported the TAC of Uslu oil was 123.67 ± 6.23 mg TE·kg^−1^, lower than that of EVOO from the same variety investigated in this study. Previously, Borges et al. (2019) [49] studied the antioxidant properties (based on DPPH assay) of EVOOs from Hojiblanca and Arbequina olives and found values between 167.69 and 352.91 mg TE·kg^−1^ and 177.70 and 380.44 mg TE·kg^−1^, respectively, in agreement with those found in this study. Reboredo-Rodríguez et al. (2018) [50] found the TAC based on the DPPH scavenging activity of EVOO from Brava olives to be 577.91 mg TE·kg^−1^. This value is similar to the TAC of the Edincik oil but higher than that of oils from other varieties.

### 2.6. Volatile Compounds (VCs)

The flavor of OO is a remarkable feature to evaluate their quality and is associated with VCs. These VCs are mostly formed during oil extraction and also develop during harvest and storage processes. The positive attributes and organoleptic defects in sensory perception of OO are associated with VCs [51]. Table 4 shows the amount of each VC and their contents of chemical classes in samples. A total of 38 volatile aroma compounds were identified in the EVOO samples. The chemical classes of these VC were as follows: terpenoids (8), alcohols (10), aldehydes (11), esters (2), acids (2), and miscellaneous (5). Almost all of the VCs detected in the EVOO samples have also been reported in many previous studies on OOs. The total concentration of VC in Edincik (83.754 mg·kg^−1^), Domat (85.077 mg·kg^−1^), Uslu (87.987 mg·kg^−1^), and Gemlik (85.044 mg·kg^−1^) oils were not different significantly from each other (*p* > 0.05) but were higher than that of Ayvalık (52.368 mg·kg^−1^) oil (*p* < 0.05).

The predominant VCs in the samples were C6 and C5 volatiles derived by lipoxygenase (LOX) pathway from linoleic and linolenic acids as unsaturated fatty acids. These VCs identified in oils were as follows: C6 aldehydes (hexanal, 3-hexenal, (E)-2-hexenal and 2,4-hexanedienal), C6 alcohols (1-hexanol, (Z)-3-hexen-1-ol and (E)-2-hexen-1-ol), C5 aldehydes (pentanal, (E)-2-pentenal) and C5 alcohols (1-penten-3-ol, 1-pentanol and (Z)-2-penten-1-ol). These compounds formed by LOX have been also reported previously as the most common volatiles in EVOO and the most responsible for their characteristic aroma [5,6,52]. In addition to these VCs, the two identified esters (hexyl acetate and (E)-3-hexen-1-ol acetate) can be formed sequentially by the LOX pathway. These esters are also associated with the typical green and fruity aroma of EVOO [53].

**Figure 2:**
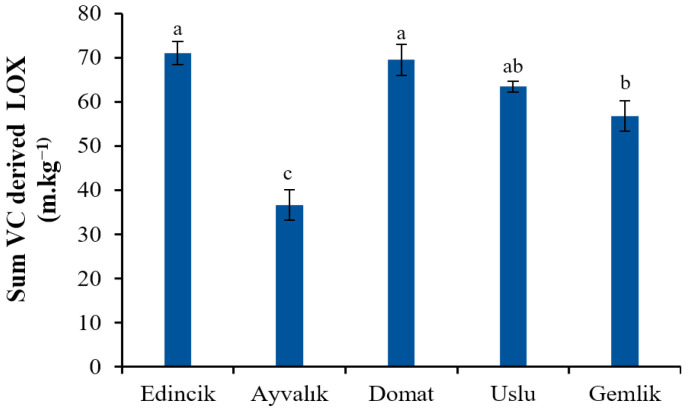
Total amount of VC derived by LOX in EVOOs from five Turkish olive cultivars. Different letters on the bars indicate significant differences between different oil samples (*p* < 0.05).

From the quantitative point of view, the total level of LOX group VC accounted for 65.87%, 21.01%, 70.49%, 57.34%, and 42.46% of the total concentration VC in Edincik, Ayvalık, Domat, Uslu, and Gemlik oils, respectively. Similar results were observed in most of the studies on the VCs of OOs. For instance, the study of Cecchi et al. (2022) [5] revealed that most of the VCs found in 320 VOO samples produced from 9 olive varieties from different geographical areas were compounds derived by LOX, as already reported in other previous papers [35,44]. It was stated that a rich C6 volatiles (both aldehydes and alcohols) content increases the intensity of the desirable green odor of OO [44,54]. The Edincik and the Domat oils had higher total amounts of C6 compounds (71.301 and 69.798 mg·kg^−1^, respectively) than those of the others, while the Ayvalık oil showed the lowest ones (36.886 mg·kg^−1^) (Figure 2). Similarly, Ilyasoglu et al. (2011) [19] showed that C6 volatiles were the most abundant VCs in both Ayvalık and Memecik EVOOs they studied.

Within C6 compounds, (E)-2-hexenal (green leaf- and almond-like) was the major compound found in all EVOO samples. (E)-2-hexenal and 3-hexenal have been reported to significantly influence the sensory properties of OO, providing its freshness and pleasant aroma [5,55]. (E)-2-hexenal only represented approximately 58%, 18%, 64%, 51% and 40% of the total VC content of Edincik, Ayvalık, Domat, Uslu and Gemlik oils, respectively. Similar percentages of this volatile compound were also obtained by several studies for EVOOs from different countries, such as Croatia [44], Portugal [55], Tunisia [35], Turkey [56], and some others [5]. On the contrary, in a study conducted by Baccouri et al. (2022) [46], (E)-2-hexenal was not found in 2 out of 5 VOO samples from Tunisia.

The amount of 1-penten-3-ol, as an aroma-active compound (grassy, green plants), was higher in Gemlik oil (15.681 mg·kg^−1^) than that of others (ranging from 0 to 8.797 mg·kg^−1^). In a previous study [57], the amount of this compound in Gemlik oil was also found to be higher than that of Ayvalık oil, as found in this study. 5-Ethyl-2(5H)-furanone (tomato-like), probably derived by autoxidation from (Z)-3-hexenal [58], was not detected in Gemlik oils. C7-C10 aldehyde VCs, including nonanal and (E)-2-decenal, were found in low amounts in the samples. These compounds are responsible for sensory defects in OOs [53].

It is reported that the effect of variety plays a key role in the variability of VCs in OOs [53]. However, the harvest time [59], conditions during and after the extraction process (i.e., temperature and time) [22,56], agronomic factors [21], planting area or geographical origin [46], and selected method of analysis [3,6] also affect the distribution of VC in OOs.

### 2.7. Sensory Attributes

**Figure 3:**
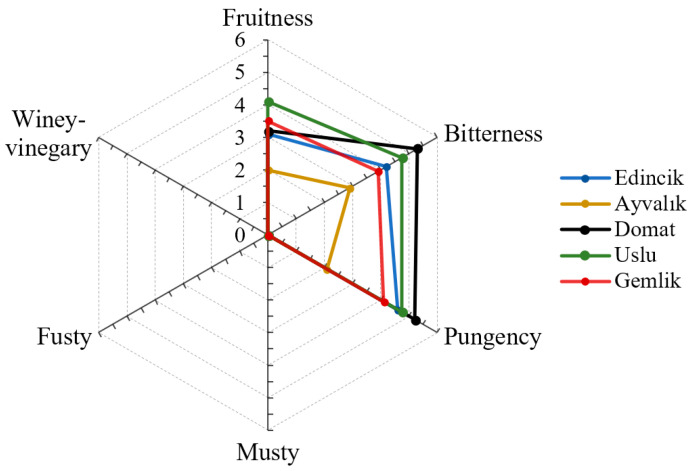
Scores of sensory attributes of EVOOs from five Turkish olive cultivars.

The results of the sensory evaluation of EVOO samples are depicted in Figure 3. The sensory attributes of all EVOO were found to be typical for EVOO (fruitiness medians > 0; three defects medians = 0) category (IOC, 2018) [60]. The highest intensity of fruitiness was determined in the EVOO of the Uslu variety, whereas that of the Ayvalık variety was characterized by the lowest intensity of the three positive sensations (*p* < 0.05). The highest scores for the perception for bitterness and pungency were observed for Domat oil (*p* < 0.05). Concerning the relationships between the positive attributes, as expected, all three perceptions strongly correlated with each other (Pearson’s coefficients ranged between 0.845 and 0.910, *p* < 0.001). Furthermore, significant correlations were found between the positive attributes and both TPC and the total concentration of the VC group synthesized by the LOX pathway. These correlations are supported by the findings of Klisović et al. (2022) [44]. Similarly, Caporale et al. (2004) [61] also reported a linear relationship between TPC and bitterness for OOs. The pungency scores of samples were highly correlated with their TPC (Pearson’s coefficients = 0.910, *p* < 0.001) and the total concentration of VC derived by LOX (Pearson’s coefficients = 0.935, *p* < 0.001). Additionally, there was also a good correlation between these VC contents (total) and fruitiness (Pearson’s coefficients = 0.647, *p* < 0.05). These results are in agreement with those discussed in several previous studies [4,17,44]. It was reported that the bitter taste in OOs is attributable to compounds oleuropein aglycone forms, while their pungency is associated with ligstroside aglycone compounds [17].

### 2.8. Principal Component Analysis (PCA)

**Figure 4:**
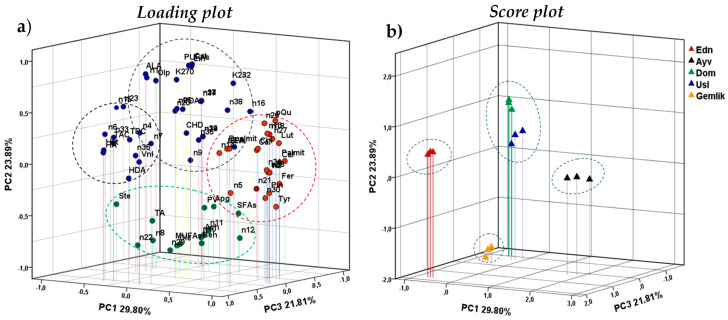
Loading plot (**a**) and score plot (**b**) of the three first components of PCA based on analyzed parameters of samples. Abbreviations: Edn, Edincik; Ayv, Ayvalık; Dom, Domat; Usl, Uslu; FFA, Free fatty acid; MUFA, Monounsaturated fatty acid; PUFA, Polyunsaturated fatty acid; SFA, Saturated fatty acid; PV, Peroxide value; TAC, Total antioxidant capacity; TPC, Total phenolic content; ALA, linolenic acid; PDA, Pentadecanoic acid; Palmit, Palmitic acid; Dpalmit, 9-Hexadecenoic acid; HDA, Heptadecanoic acid, CHD, 10-Heptadecanoic acid, Ste, Stearic acid; Ole, Oleic acid; Lin, Linoleic Acid, Ach, Eicosanoic acid; CEA, 11-Eicosenoic acid; HA, Heneicosanoic acid; Beh, Docosanoic acid; n, Compound numbered from 1 to 38 in Table 4. The components/properties in the red, green, and black cycles in the Loading plot contributed to the separation of Ayvalık, Gemlik and Edincik oils, respectively, while those in the blue circle contributed to the separation of Domat and Uslu oils from others.

PCA was applied to all data sets (except for sensory properties) to visually reveal the relationships between the variables and the samples. The results of PCA are illustrated in Figure 4. The first three principal components (PCs) accounted for 75.50% of the total variance of the data set. PC1, PC2, and PC3 explained 28.90%, 23.89%, and 21.81% of the total variance, respectively. In PCA, the first five PCs with eigenvalues greater than 1 were obtained. However, only the first three were used for its evaluation as they could explain most of the total variance. The factor loading values and eigenvalues are given in the supplementary material (SM) (Table S1).

Figure 4 shows that PC1 differentiates the Ayvalık oil from the oils of other varieties according to higher VC (β-sesquiphellandrene, pyran aldehyde, α-bergamotene, (E)-3-hexen-1-ol acetate, hexyl acetate, etc.) and PC (tyrosol, *t*-ferulic acid, caffeic acid, pinoresinol, etc.) contents (Figure 4a,b). Similarly, this oil separated from the other samples due to its lower TPC and (E)-2-hexenal concentration. Although Uslu, Domat, and Edincik oils separated from the other samples along PC2, the first two are located closer to each other on the score plot because of their similar chemical compositions, and the latter (Edincik) differs by PC3. Edincik oil is characterized predominantly by higher hydroxtyrosol, heptadecanoic acid, α-muurolene, decanoic acid, and heneicosanoic acid. Gemlik oil discriminated from other samples along PC2 mainly based on its lower linoleic acid PUFAs contents and higher oleic acid, 3-hexenal, MUFAs, and 1-penten-3-ol contents.

## 3. Materials and Methods

### 3.1. Samples

**Figure 5:**
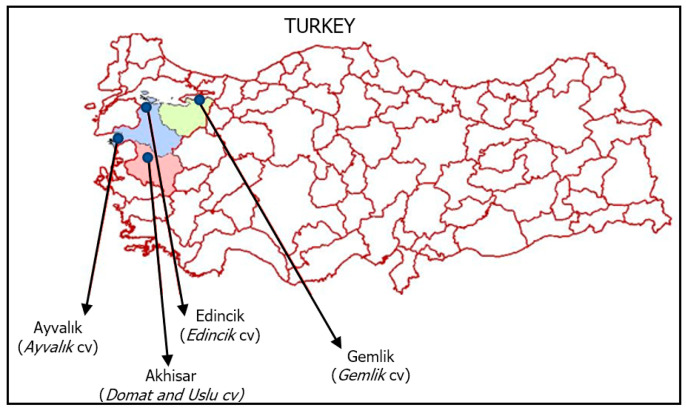
The geographical distribution of olive cultivars used for oil samples.

The samples of monovarietal EVOO used were obtained from olive cultivars of Aegean and Marmara regions (Turkey): *Edincik* (Edincik, Marmara), *Ayvalık* (Ayvalık, Marmara), *Domat* (Akhisar, Aegean), *Uslu* (Akhisar, Aegean) and *Gemlik* (synonym *Trilye*) (Gemlik, Marmara) (Figure 5) The morphological and quality characteristics [62] and phenotypes [63] of the olive varieties are given in the SM (Table S2 and Figure S1). About 300 kg of olive fruits of each cultivar were harvested in the last week of October of the crop season 2020/2021. Their maturities indices were between 1.0 and 1.2, (yellow-green) as reported by IOC (2011) [64]. These olive fruits of cultivars were processed separately with a two-phase system (MORI-TEM srl 1000 3GV 400, Florence, Italia) within 8 h after harvest to obtain the EVOO samples. The temperature and time of malaxation were 25 ± 1 °C and 30 min, respectively. After the extraction, 1 L of each EVOO was stored in dark glass bottles (100 mL) at −20 °C until analysis. For each olive cultivar used, oil extraction and all analyses were performed in triplicate.

### 3.2. Chemicals

Sodium hydroxide, sodium carbonate, potassium chromate, potassium iodide, phosphoric acid, methanol, ethanol, acetic acid (glacial), diethyl ether, phenolphthalein, chloroform, sodium thiosulfate, cyclohexane, acetonitrile, Folin–Ciocalteu reagent and standards of oleuropein, hydroxytyrosol, tyrosol, pinoresinol, *p*-qumaric acid, caffeic acid, syringic acid, vanillic acid, ferulic acid, gallic acid, luteolin, apigenin, isobutyl acetate, DPPH, Trolox and fatty acid methyl esters (FAMEs) mixture were purchased from Sigma-Aldrich (Darmstadt, Germany).

### 3.3. Quality Parameters Analysis

The FFA, PV, and extinction coefficients (K_270_ and K_270_) of the samples were determined according to the Turkish Official Methods (2014) [65]. FFA and PV were expressed as % oleic acid and meq O_2_·kg^−1^, respectively.

### 3.4. Extraction of Phenolic Compounds (PC)

The extraction of PC in the samples was carried out based on the method of IOC (2017) [66], as described by Rodrigues et al. (2019) [67], with slight modifications. Measures of 3 g of EVOO and 250 µL of syringic acid solution (0.15 mg·mL^−1^) prepared in methanol:water (80:20, v.v^−1^) were mixed and shaken in a 12 mL tube. Then, 3 mL of methanol:water was added and vortexed for 30 s. Thereafter, this mixture was centrifuged at 500 rmp at 4 °C for 5 min. The lower phase was transferred to another tube and the extraction was repeated two more times. Then, the collected methanolic phases were washed with 1.5 hexane two times to remove the oil residues. The lower phase was taken and used as the phenolic extract for the analysis of TPC, TAC, and PC.

### 3.5. Total Chlorophyll and Carotenoid Content Analysis

The total amounts of chlorophyll and carotenoid in samples were determined according to the method of Mínguez-Mosquera et al. (1991) [30]. Briefly, 7.5 g of EVOO was weighed in a tube and the volume was adjusted to 25 mL with cyclohexane, followed by vortexing for one minute. Then, the absorbances of this mixture were measured with a Biochrom Libra S70 Dual UV–vis spectrophotometer (Harvard Bioscience Co. Shanghai, China) at 470 nm and 670 nm against cyclohexane for chlorophylls and carotenoids, respectively. The total chlorophyll and carotenoid contents were calculated using the following Equations (1) and (2) and expressed as mg·kg^−1^ of pheophytin and lutein, respectively.

Carotenoids = (Abs_470_ × 106) / (2000 × 100 × d) (1)

Chlorophyl = (Abs_670_ × 106) / (613 × 100 × d) (2)

where Abs_470_ and Abs_670_ are the absorbance values read at these wavelengths, and d is the length of the optical path (1 cm).

### 3.6. Total Phenolic Content (TPC) Analysis

The TPCs of samples were determined by using the Folin–Ciocalteu method, as adopted by Capanoglu et al. (2013) [68]. Measures of 100 µL of the phenolic extract, 900 µL of deionized water, and 5 mL of Folin–Ciocalteu reagent (0.2 N) were mixed in a tube and kept for 8 min. Then, 5 mL of sodium carbonate was added and vortexed for 30 s. This mixture was left in the dark at the room temperature for 2 h and then its absorbance was measured with a UV–vis spectrophotometer (Biochrom Libra S70 Dual) at 765 nm. The results were calculated from a calibration curve created with different solutions of gallic acid as standard and expressed as mg gallic acid equivalent (GAE) per kg of samples.

### 3.7. Total Antioxidant Capacity (TAC) Analysis

The TAC of the samples was determined using the DPPH radical scavenging capacity of the metabolic extracts based on the method reported by Osei et al. (2022) [69], with some modifications. A measure of 3 mL of 60 µM DPPH in methanol was added to 0.5 mL of the phenolic extract and the resulting mixture was incubated in dark for 30 min at room temperature. Then, the absorbance of this solution was recorded at 517 nm against methanol as the blank using a UV–vis spectrophotometer (Biochrom Libra S70 Dual). For the control sample, a methanol:water solution (80:20 *v*/*v*) was used instead of the phenolic extract. The percentage of DPPH inhibition of each phenolic extract of samples was calculated following Equation (3):

DPPH inhibition (%) = (Abs_control_−Abs_sample_) / (Abs_control_) (3)

where Abs_control_ and Abs_sample_ were absorbances recorded at 517 nm for the control and phenolic extract of the sample, respectively.

The value of IC50 of each sample, which corresponds to the concentration of extract reducing half of the amount of DPPH radical, was calculated from the regression curve obtained using five different phenolic extract solutions diluted by methanol:water solution. Trolox was used as standard and the results were expressed as mg Trolox equivalent (TE) per kg sample.

### 3.8. Fatty Acid Composition Analysis

The analysis of FA composition in samples was carried out by a gas chromatography and flame ionization detector (GC-FID) system (Shimadzu QP2020, Shimadzu Corp., Kyoto, Japan) equipped with an Rtx-2330 capillary column (0.20 µm, 60 m × 0.25 mm, Restek, Bad Homburg, Germany) [65]. Approximately 0.1 g of oil sample was added to 10 mL of hexane and shaken vigorously. To obtain FAMEs, 0.5 mL of the solution of potassium hydroxide (2N) in methanol was added to this mixture and vortexed for 20 s. After holding in the dark for 2 h, 1 µL of this solution was injected into the GC with a split mode (1:100). The temperatures of the injection port and detector were set at 250 °C. The oven temperature was first set at 140 for 5 min. Then, it was increased to 240 °C at a rate of 4 °C/min and maintained at isotherm for 12 min. The carrier gas was helium at a flow rate of 1 mL/min. The peak identifications and calculation of their areas as relative percentages were performed by using the mixture standards of FAMEs.

### 3.9. Phenolic Compounds (PC) Analysis

The analysis of phenolic fractions in samples was carried out using a Water Alliance e2695 HPLC (Waters, Milford, MA, USA) system, consisting of a photodiode array detector (PDA) (Waters 2996, Milford, MA, USA) and an inertSustain C18 (5 µm, 4.6 × 250 mm, GL Sciences, Tokyo, Japan). The phenolic extract was filtered through a 0.45 μm polyvinylidine fluoride (PVDF) syringe filter before the injection into the system. The operational procedures of the HPLC were performed as described by Veneziani et al. (2018) [8], with some modifications. The results are expressed as mg·kg^−1^. Details of the analysis are presented in the SM.

### 3.10. Volatile Compound (VC) Analysis

The VCs in samples were isolated by solid phase micro-extraction (SPME) and analyzed with a gas chromatography-mass detector (GC-MS) (Shimadzu QP2020, Shimadzu Corp., Kyoto, Japan) system coupled to an autosampler (AOC 5000 Plus, CTC, Switzerland), according to Genovese et al. (2015) [54] and Korkmaz et al. (2020) [70], with some modifications. Details of the analysis are presented in SM.

### 3.11. Sensory Analysis

The sensory properties of samples were evaluated by a trained sensory panel of ten assessors from the Central Laboratory of Mardin Artuklu University (Mardin, Turkey) according to the Turkish Official Methods (2014) [65] adapted from the procedure of IOC (2018) [60]. The quantitative intensity of positive attributes (fruitiness, bitterness, and pungency) and defects (musty, fusty, winey–vinegary) were determined by marking the scale from 0 (no perception) to 10 (the highest intensity) cm on the original profile sheet of the method used. The results were expressed as median values of assessor perception scores.

### 3.12. Statistical Analysis

The significance of differences among the values of all parameters of samples was determined by one-way analysis of variance (ANOVA) followed by Duncan’s multi-comparison test (*p* < 0.05). Principal component analysis (PCA) as a multivariate analysis was also performed to compare the sample based on their investigated properties. All statistical analyses were carried out using the SPSS (version 16.0, Chicago, IL, USA) software package.

## 4. Conclusions

The monovarietal EVOOS from five different Turkish olive varieties were characterized in terms of their FA, PC, and VC profiles as well as TPC, TAC, pigment contents, and sensory properties. Additionally, the EVOO samples were compared with each other based on these properties. There were significant differences between the major components in FA, PC, and VC profiles of the oil samples. The highest TAC was obtained for the EVOO from the Edincik variety, possibly due to it having the highest hydroxytyrosol content. The Domat oil exhibited the highest TPC and, therefore, probably also had higher scores for bitterness and pungency. The scores obtained for the fruitiness perceptions of the oils (except for the Ayvalık oil) were close to each other. This was also confirmed by the relationship between their total content of VCs derived from LOX. The result of PCA showed that the EVOO from Ayvalık olives is distinct from the oils from other varieties due to its lower values for many quality attributes. Based on this study, it can be concluded that the EVOO from Domat olives had the best characteristics sensory quality parameters and chemical properties affecting them. In the future, studies should be conducted on the storage stability of these oils.

## Figures and Tables

**Table 1:** Legal quality parameters and pigment contents of EVOOs from five Turkish olive cultivars.

Parameter	Edincik	Ayvalık	Domat	Uslu	Gemlik
FFA (% oleic acid)	0.09 ± 0.01 ^b^	0.12 ± 0.01 ^a^	0.06 ± 0.00 ^c^	0.06 ± 0.01 ^c^	0.05 ± 0.00 ^c^
PV (meq O_2_·kg^−1^)	5.23 ± 0.79 ^ab^	6.24 ± 0.23 ^a^	5.45 ± 0.16 ^ab^	3.50 ± 0.27 ^c^	6.33 ± 0.72 ^a^
K_232_	1.78 ± 0.04 ^c^	1.89 ± 0.01 ^b^	1.99 ± 0.02 ^a^	1.74 ± 0.02 ^c^	1.64 ± 0.01 ^d^
K_270_	0.18 ± 0.02 ^a^	0.16 ± 0.01 ^b^	0.16 ± 0.01 ^b^	0.18 ± 0.01 ^a^	0.13 ± 0.00 ^c^
Chlorophylls (mg·kg^−1^)	3.60 ± 0.12 ^c^	5.52 ± 0.01 ^a^	1.92 ± 0.16 ^d^	4.53 ± 0.11 ^b^	1.84 ± 0.12 ^d^
Carotenoids (mg·kg^−1^)	1.99 ± 0.04 ^b^	4.39 ± 0.01 ^a^	1.59 ± 0.06 ^c^	4.47 ± 0.02 ^a^	1.67 ± 0.05 ^c^
Total pigments (mg·kg^−1^)	5.58 ± 0.16 ^c^	9.90 ± 0.02 ^a^	3.51 ± 0.22 ^d^	9.00 ± 0.13 ^a^	3.51 ± 0.18 ^d^

Abbreviations: SFA, Saturated fatty acids; PUFA, Polyunsaturated fatty acids; MUFA, Monounsaturated fatty acids. ^a–d^ Means with different lowercase letters in the same row indicate significant differences between cultivars (*p* < 0.05).

**Table 2:** Fatty acid profile (%) of EVOOs from five Turkish olive cultivars.

Fatty Acid	Edincik	Ayvalık	Domat	Uslu	Gemlik
Oleic acid (C18:1)	69.73 ± 0.04 ^d^	70.21 ± 0.02 ^b^	70.08 ± 0.03 ^c^	67.87 ± 0.05 ^e^	74.13 ± 0.01 ^a^
Palmitic acid (C16:0)	12.88 ± 0.01 ^e^	14.58 ± 0.01 ^a^	14.10 ± 0.02 ^c^	14.51 ± 0.02 ^b^	13.84 ± 0.01 ^d^
Linoleic Acid (C18:2)	10.80 ± 0.06 ^b^	9.47 ± 0.02 ^d^	10.10 ± 0.02 ^c^	11.11 ± 0.02 ^a^	5.43 ± 0.02 ^e^
Stearic acid (C18:0)	2.98 ± 0.01 ^a^	2.26 ± 0.00 ^d^	1.96 ± 0.01 ^e^	2.53 ± 0.01 ^c^	2.74 ± 0.01 ^b^
Linolenic acid (C18:3)	0.94 ± 0.01 ^b^	0.68 ± 0.01 ^b^	0.94 ± 0.01 ^a^	0.95 ± 0.02 ^a^	0.68 ± 0.01 ^a^
9-Hexadecenoic acid (C16:1)	0.74 ± 0.01 ^d^	0.88 ± 0.00 ^c^	1.10 ± 0.00 ^a^	0.93 ± 0.03 ^b^	0.96 ± 0.01 ^b^
Heptadecanoic acid (C17:0)	0.20 ± 0.01 ^a^	0.15 ± 0.02 ^b^	0.12 ± 0.01 ^b^	0.15 ± 0.01 ^b^	0.15 ± 0.01 ^b^
10-Heptadecanoic acid (C17:1)	0.26 ± 0.01 ^a^	0.25 ± 0.00 ^a^	0.24 ± 0.01 ^b^	0.25 ± 0.01 ^ab^	0.23 ± 0.00 ^b^
Eicosanoic acid (C20:0)	0.40 ± 0.06 ^ab^	0.44 ± 0.01 ^a^	0.34 ± 0.00 ^b^	0.44 ± 0.01 ^a^	0.46 ± 0.01 ^a^
11-Eicosenoic acid (C20:1)	0.30 ± 0.04 ^a^	0.32 ± 0.00 ^a^	0.29 ± 0.01 ^a^	0.28 ± 0.00 ^a^	0.28 ± 0.01 ^a^
Docosanoic acid (C22:0)	0.12 ± 0.00 ^a^	0.13 ± 0.00 ^a^	0.10 ± 0.00 ^b^	0.12 ± 0.00 ^a^	0.13 ± 0.01 ^a^
Tricosanoic acid (C23:0)	0.62 ± 0.01 ^c^	0.52 ± 0.01 ^e^	0.56 ± 0.01 ^d^	0.76 ± 0.01 ^b^	0.82 ± 0.01 ^a^
SFAs	17.27 ± 0.05 ^c^	18.15 ± 0.03 ^b^	17.25 ± 0.01 ^c^	18.57 ± 0.01 ^a^	18.23 ± 0.03 ^b^
PUFAs	11.73 ± 0.05 ^b^	10.15 ± 0.03 ^d^	11.03 ± 0.01 ^c^	12.05 ± 0.03 ^a^	6.10 ± 0.02 ^e^
MUFAs	71.02 ± 0.01 ^c^	71.67 ± 0.02 ^b^	71.71 ± 0.02 ^b^	69.34 ± 0.02 ^d^	75.61 ± 0.03 ^a^
MUFAs/PUFAs	6.06 ± 0.025 ^d^	7.07 ± 0.015 ^b^	6.51 ± 0.005 ^c^	5.76 ± 0.015 ^e^	12.4 ± 0.03 ^a^

Abbreviations: SFA, Saturated fatty acids; PUFA, Polyunsaturated fatty acids; MUFA, Monounsaturated fatty acids. ^a–e^ Means with different lowercase letters in the same row indicate significant differences between cultivars (*p* < 0.05).

**Table 3:** Phenolic compounds (mg·kg^−1^) in EVOOs from five Turkish olive cultivars.

Phenolic Compound	Edincik	Ayvalık	Domat	Uslu	Gemlik
Hydroxytyrosol	48.022 ± 1.186 ^a^	11.621 ± 0.420 ^c^	11.646 ± 0.229 ^c^	15.679 ± 1.735 ^b^	18.165 ± 0.592 ^b^
Oleuropein	10.351 ± 1.139 ^b^	2.317 ± 0.235 ^c^	10.604 ± 0.642 ^b^	13.722 ± 0.416 ^a^	2.415 ± 0.239 ^c^
Pinoresinol	6.899 ± 0.366 ^d^	15.247 ± 0.524 ^a^	7.265 ± 0.355 ^d^	13.263 ± 0.376 ^b^	10.499 ± 0.837 ^c^
Apigenin	2.409 ± 0.030 ^e^	6.521 ± 0.212 ^c^	11.034 ± 0.204 ^b^	4.679 ± 0.348 ^d^	12.824 ± 0.010 ^a^
Luteolin	1.640 ± 0.089 ^c^	2.658 ± 0.080 ^a^	2.358 ± 0.050 ^b^	1.833 ± 0.107 ^c^	1.850 ± 0.043 ^c^
Tyrosol	1.403 ± 0.023 ^d^	3.500 ± 0.109 ^a^	1.887 ± 0.066 ^c^	2.017 ± 0.020 ^c^	2.477 ± 0.111 ^b^
Vanillic acid	1.991 ± 0.031 ^a^	1.703 ± 0.045 ^ab^	1.550 ± 0.037 ^b^	1.445 ± 0.220 ^b^	1.617 ± 0.024 ^ab^
*p*-Qumaric acid	0.079 ± 0.006 ^b^	0.715 ± 0.005 ^a^	0.644 ± 0.011 ^a^	0.797 ± 0.152 ^a^	0.233 ± 0.005 ^b^
*t*-Ferrulic acid	0.095 ± 0.020 ^e^	0.944 ± 0.016 ^a^	0.194 ± 0.006 ^d^	0.537 ± 0.028 ^b^	0.342 ± 0.034 ^c^
Catechin	0.440 ± 0.020 ^d^	0.294 ± 0.025 ^a^	0.623 ± 0.033 ^c^	0.644 ± 0.036 ^b^	0.086 ± 0.005 ^c^
Caffeic acid	0.009 ± 0.001 ^d^	0.184 ± 0.002 ^a^	0.033 ± 0.001 ^c^	0.129 ± 0.001 ^b^	0.034 ± 0.001 ^c^
TPC	294.090 ± 13.810 ^c^	234.71 ± 1.490 ^d^	350.6 ± 8.770 ^a^	291.145 ± 5.485 ^c^	321.905 ± 6.185 ^b^

Abbreviations: SFA, Saturated fatty acids; PUFA, Polyunsaturated fatty acids; MUFA, Monounsaturated fatty acids. ^a–e^ Means with different lowercase letters in the same row indicate significant differences between cultivars (*p* < 0.05).

**Table 4:** Composition of volatile compounds (mg·kg^−1^) in EVOOs from five Turkish olive cultivars.

No	Volatile Compound	RI ^1^	Edincik	Ayvalık	Domat	Uslu	Gemlik
	Terpenoids		7.584 ^a^	3.8905 ^b^	3.218 ^b^	3.765 ^b^	3.994 ^b^
1	(Z)- β-Ocimene	1245	1.642 ^a^	0.183 ^b^	1.534 ^a^	1.707 ^a^	0.129 ^b^
2	α-Bergamotene	1596	nd	1.029 ^a^	nd	nd	nd
3	β-Sesquiphellandrene	1730	nd	0.462 ^a^	nd	nd	nd
4	α-Muurolene	1741	0.692 ^a^	0.259 ^b^	nd	0.174 ^b^	nd
5	α-Curcumene	1788	0.264 ^ab^	0.627 ^a^	nd	nd	0.214 ^ab^
6	α -Copaene	1506	3.056 ^a^	0.391 ^c^	0.628 ^c^	1.024 ^b^	0.713 ^bc^
7	Cyclosativene	1499	0.887 ^a^	0.440 ^b^	nd	nd	nd
8	(E)-4,8-Dimethyl-1,3,7-nonatriene	1322	1.042 ^b^	0.499 ^a^	1.056 ^b^	0.860 ^bc^	2.938 ^a^
	Alcohols		7.455 ^c^	14.391 ^b^	11.713 ^bc^	15.995 ^b^	23.793 ^a^
9	Ethanol	993	nd	nd	0.238 ^a^	0.096 ^bc^	0.175 ^ab^
10	1-Penten-3-ol	1179	nd	3.930 ^c^	4.162 ^c^	8.797 ^b^	15.681 ^a^
11	1-Pentanol	1243	2.351 ^b^	3.278 ^ab^	2.416 ^ab^	4.182 ^ab^	4.933 ^a^
12	(Z)-2-Penten-1-ol	1331	0.450 ^c^	1.013 ^ab^	0.681 ^bc^	0.858 ^ab^	1.259 ^a^
13	1-Hexanol	1361	0.530 ^c^	1.466 ^a^	0.792 ^b^	0.438 ^c^	0.250 ^d^
14	(Z)-3-Hexen-1-ol	1392	3.061 ^b^	3.920 ^a^	1.574 ^c^	1.625 ^c^	1.495 ^c^
15	(E)-2-Hexen-1-ol	1413	1.062 ^a^	nd	0.517 ^b^	nd	nd
16	2-Ethylhexanol	1492	nd	0.383 ^b^	0.582 ^a^	nd	nd
17	Phenylmethanol	1884	nd	nd	0.484 ^a^	nd	nd
18	Phenylethanol	1922	nd	0.402 ^a^	0.267 ^b^	nd	nd
	Aldehydes		67.029 ^ab^	25.788 ^c^	68.165 ^a^	61.976 ^ab^	55.620 ^b^
19	Pentanal	1030	nd	nd	nd	0.401 ^a^	nd
20	Hexanal	1080	3.939 ^ab^	3.218 ^bc^	2.864 ^c^	4.180 ^a^	2.362 ^c^
21	(E)-2-Pentenal	1130	0.220 ^a^	0.343 ^a^	0.265 ^a^	0.343 ^a^	0.313 ^a^
22	3-Hexenal	1132	11.026 ^b^	7.248 ^c^	6.609 ^c^	9.152 ^bc^	17.237 ^a^
23	(E)-2-Hexenal	1228	48.964 ^b^	9.558 ^d^	54.485 ^a^	44.874 ^b^	33.647 ^c^
24	(E)-2-Heptenal	1341	nd	nd	0.402 ^a^	nd	nd
25	Nonanal	1408	0.540 ^a^	0.386 ^a^	0.350 ^a^	nd	nd
26	2,4-Hexanedienal	1413	2.339 ^ab^	4.160 ^a^	2.958 ^ab^	3.025 ^ab^	1.703 ^b^
27	Benzaldehyde	1541	nd	0.382 ^a^	0.232 ^b^	nd	nd
28	Pyran aldehyde	1611	nd	0.493 ^a^	nd	nd	nd
29	(E)-2-Decenal	1658	nd	nd	nd	nd	0.358 ^a^
	Esters		0.388 ^b^	7.3185 ^a^	nd	0.378 ^b^	0.357 ^b^
30	Hexyl acetate	1291	nd	1.282 ^a^	nd	nd	0.356 ^b^
31	(E)-3-Hexen-1-ol acetate	1334	0.388 ^b^	6.036 ^a^	nd	0.378 ^b^	nd
	Acids		0.144 ^a^	0.188 ^a^	nd	nd	nd
32	Octanoic acid	2094	nd	0.188 ^a^	nd	nd	nd
33	Decanoic acid	2228	0.144 ^a^	nd	nd	nd	nd
	Miscellaneous		1.156 ^b^	0.794 ^b^	1.981 ^b^	5.872 ^a^	1.281 ^b^
34	Methylbenzene		nd	nd	nd	0.536 ^a^	0
35	3-Ethyl-1,5-octadiene	1053	0.715 ^ab^	0.400 ^bc^	0.770 ^a^	0.278 ^c^	0.652 ^ab^
36	1,2-Dimethyl benzene	1183	0.181 ^b^	nd	0.641 ^b^	4.805 ^a^	0.629 ^b^
37	Pyridine	1194	nd	nd	0.200 ^a^	nd	nd
38	5-Ethyl-2(5H)-furanone	1610	0.260 ^a^	0.394 ^a^	0.370 ^a^	0.252 ^a^	nd
	Total		83.754 ^a^	52.368 ^b^	85.077 ^a^	87.987 ^a^	85.044 ^a^

^1^ Calculated on DB-HeavyWax column. Abbreviations: RI, Retention indices. ^a–d^ Means with different lowercase letters in the same row indicate significant differences between cultivars (*p* < 0.05).

## Data Availability

Not applicable.

## Supplementary Materials

The following supporting information can be downloaded at: https://www.mdpi.com/article/10.3390/molecules28031483/s1, Table S1: Rotated factor loadings, eigenvalues and variances explained of the three first principal components; Table S2: Morphological and quality characteristics of the olive plant/fruit variety used in the study; Figure S1: Fruits, leaves and seeds of olive varieties used.

## Sample Availability

Samples of the compounds are not available from the author.
