# Supplementary material for: Characterization and Comparison of Extra Virgin Olive Oils of Turkish Olive Cultivars
Source: Molecules. 2023 Feb 3;28(3):1483. doi: 10.3390/molecules28031483 (PMC9919864; doi:10.3390/molecules28031483)
Supplement: Supplementary file 1 [file molecules-28-01483-s001.zip › molecules-2168492-supplementary.pdf]

Table S1. Rotated factor loadings, eigenvalues and variances explained of the three first principal components

| Variables | Component | | | Variables | Component | | |
|---|---|---|---|---|---|---|---|
| | PC1 | PC2 | PC3 | | PC1 | PC2 | PC3 |
| β-Sesquiphellandrene | **0.985** | -0.034 | 0.093 | Heptadecanoic acid | -0.115 | -0.082 | **0.909** |
| Pyran aldehyde | **0.985** | -0.034 | 0.095 | α-Muurolene | 0.014 | 0.369 | **0.908** |
| α-Bergamotene | **0.981** | -0.035 | 0.090 | Hydroxtyrosol | -0.420 | 0.165 | **0.889** |
| (E)-3-Hexen-1-ol acetate | **0.976** | -0.007 | 0.129 | Decanoic acid | -0.326 | 0.293 | **0.858** |
| Octanoic acid | **0.971** | -0.032 | 0.098 | Heneicosanoic acid | -0.449 | 0.135 | **0.855** |
| Hexyl acetate | **0.918** | -0.291 | 0.067 | Vanilic acid | -0.032 | 0.076 | **0.851** |
| Trans-ferrulic acid | **0.916** | -0.182 | -0.183 | Cyclosativene | 0.092 | 0.262 | **0.828** |
| 1-Hexanol | **0.891** | 0.325 | -0.009 | a-Copaene | -0.453 | 0.309 | **0.821** |
| Caffeic acid | **0.872** | 0.014 | -0.250 | Stearic acid | -0.356 | -0.369 | **0.765** |
| Tyrosol | **0.864** | -0.411 | -0.201 | 10-Heptadecanoic acid | 0.398 | 0.370 | **0.667** |
| Benzaldehyde | **0.792** | 0.239 | -0.243 | Total phenolic content | **-0.898** | 0.095 | -0.269 |
| Free fatty acid | **0.788** | 0.229 | 0.496 | (E)-2-Hexenal | **-0.867** | 0.437 | -0.114 |
| Pinoresinol | **0.772** | -0.287 | -0.216 | Tricosanoic acid | **-0.540** | -0.648 | -0.132 |
| Chlorophyll | **0.761** | 0.165 | -0.039 | 3-Ethyl-1,5-octadiene | **-0.521** | 0.023 | 0.188 |
| Luteolin | **0.753** | 0.173 | -0.415 | Ethanol | **-0.506** | -0.129 | -0.742 |
| 4-Hexanedienal | **0.747** | 0.394 | -0.172 | (E)-2-Decenal | -0.290 | **-0.907** | -0.071 |
| Phenylethanol | **0.743** | 0.277 | -0.265 | Oleic acid | -0.148 | **-0.840** | 0.022 |
| (Z)-3-Hexen-1-ol | **0.738** | 0.195 | 0.599 | 3-Hexenal | -0.445 | **-0.833** | 0.263 |
| α-Curcumene | **0.736** | -0.216 | 0.401 | MUFAs | -0.145 | **-0.831** | -0.029 |
| Carotenoids | **0.704** | 0.138 | -0.102 | (E)-4,8-Dimethyl-1,3,7-nonatriene | -0.494 | **-0.829** | -0.073 |
| Palmitic acid | **0.595** | -0.051 | -0.752 | 1-Penten-3-ol | -0.185 | **-0.820** | -0.453 |
| 11-Eicosenoic acid | **0.589** | 0.183 | 0.244 | (Z)-2-Penten-1-ol | 0.314 | **-0.779** | -0.386 |
| p-Qumaric acid | **0.507** | 0.336 | -0.717 | Docosanoic acid | 0.388 | **-0.734** | 0.387 |
| PUFAs | 0.049 | **0.935** | 0.109 | 1-Pentanol | 0.032 | **-0.716** | -0.262 |
| Linoleic Acid | 0.086 | **0.931** | 0.116 | Eicosanoic acid | 0.251 | **-0.695** | 0.153 |
| Catechin | -0.193 | **0.879** | -0.308 | Apigenin | -0.157 | **-0.539** | -0.632 |
| $K_{270}$ | 0.016 | **0.813** | 0.272 | SFAs | 0.352 | **-0.527** | -0.306 |
| Linolenic acid | -0.548 | **0.789** | -0.026 | 9-Hexadecenoic acid | -0.124 | 0.007 | **-0.940** |
| (Z)- β-Ocimene | -0.512 | **0.761** | 0.006 | Pyridine | -0.264 | 0.488 | **-0.576** |
| $K_{232}$ | 0.311 | **0.737** | -0.276 | (E)-2-Heptenal | -0.264 | 0.489 | **-0.575** |
| Oleuropin | -0.496 | **0.717** | -0.127 | Phenylmethanol | -0.256 | 0.484 | **-0.557** |
| Nonanal | 0.212 | **0.579** | 0.557 | 2-Ethylhexanol | 0.365 | 0.443 | -0.489 |
| Pentadecanoic acid | 0.198 | **0.559** | 0.434 | 1,2-Dimethyl benzene | -0.184 | 0.132 | -0.408 |
| (E)-2-Hexen-1-ol | -0.461 | **0.544** | 0.609 | (E)-2-Pentenal | 0.496 | -0.289 | -0.406 |
| Hexanal | 0.027 | **0.515** | 0.312 | Pentanal | -0.085 | 0.181 | -0.335 |
| 5-Ethyl-2(5H)-furanone | 0.385 | **0.506** | -0.067 | Methylbenzene | -0.086 | 0.181 | -0.335 |
| Total antioxidant capacity | -0.289 | 0.250 | **0.918** | Peroxide value | 0.254 | -0.427 | 0.141 |

| | | | |
|---|---|---|---|
| Variance explained of total varince | 29.80% | 23.89% | 21.81% |
| Sum of variance | | 75.50% | |
| Eigenvalues | 22.43 | 18.82 | 14.63 |

Table S2. Morphological and quality characteristics of the olive plant/fruit variety used in the study [62]

| Phenolic compound | Edincik | Ayvalık | Domat | Uslu | Gemlik |
|---|---|---|---|---|---|
| ***Flower bud*** | | | | | |
| Length | short | Medium | medium | medium | short |
| Number of flowers | small | medium | small | small | small |
| ***Leaf*** | | | | | |
| Shape | long-elliptical | long-elliptical | long-elliptical | long-elliptical | long-elliptical |
| Length | long | medium | medium | medium | medium |
| Width | medium | medium | medium | medium | medium |
| ***Fruit*** | | | | | |
| Weight | medium | medium | extra-large | medium | medium |
| Shape | oval | oval | elliptical | oval | oval |
| Symmetry | symmetrical | symmetrical | symmetrical | slight-symmetrical | slight-symmetrical |
| Widest point | towards tip | central | central | central | central |
| Fruit tip | round | round | round | pointed | round |
| Stem part | cut | round | round | round | cut |
| ***Stone*** | | | | | |
| Weight | medium | medium | large | large | medium |
| Shape | elliptical | oval | long | elliptical | oval |
| Symmetry | symmetrical | symmetrical | symmetrical | asymmetric | slight-symmetrical |
| Widest point | towards tip | near-end | central | central | towards tip |
| Stone tip | pointed | pointed | pointed | pointed | round |
| Stem part | pointed | cut | pointed | pointed | round |
| Surface | rough | rough | rough | rough | rough |
| Tip point | pinpoint | pinpoint | pinpoint | pinpoint | pinpoint |
| ***Quality features*** | | | | | |
| Number of fruits (kg) | 311 | 291 | 140 | 260 | 273 |
| Pulp ratio (%) | 89 | 89 | 88 | 88 | 86 |
| Stone ratio (%) | 11 | 11 | 12 | 12 | 14 |
| Oil ratio (%) | low (<18%) | high (>22%) | low (<18%) | low (<18%) | high (>22%) |

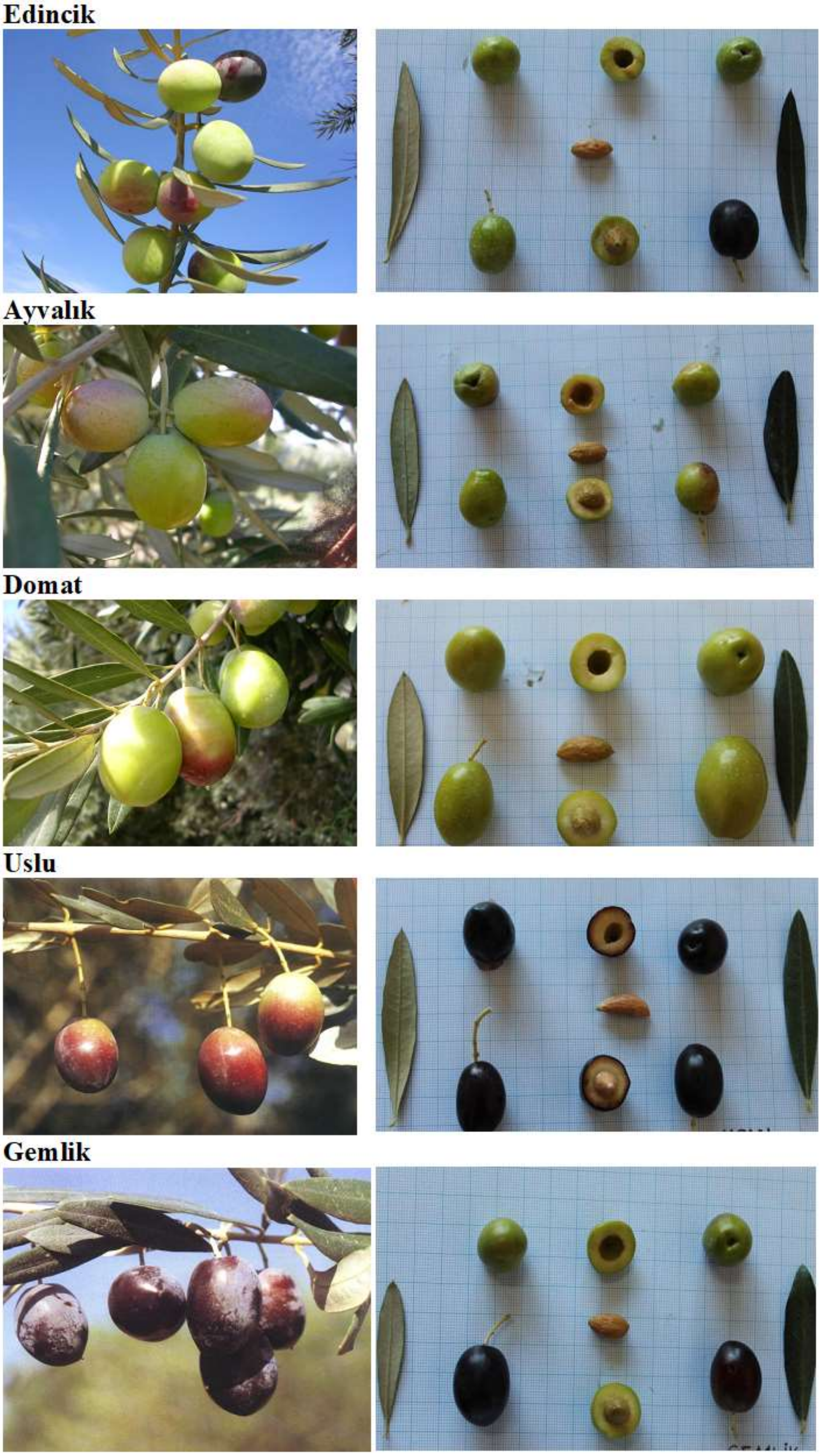


Figure S1. Fruits, leaves and seeds of olive varieties used [63]

**Materials and Methods**

*Phenolic compounds (PC) analysis*

The analysis of phenolic fractions in samples was carried out using a Water Alliance e2695 HPLC (Waters, Milford, MA, USA) system, consisting of a photodiode array detector (PDA) (Waters 2996, Milford, MA, USA) and an inertSustain C18 (5µm, 4.6 x 250 mm, GL Sciences, Tokyo, Japan). The phenolic extract was filtered through a 0.45 µm polyvinylidine fluoride (PVDF) syringe filter before the injection into the system. The operational procedures of the HPLC were performed as described by Veneziani et al. (2018) [8], with some modifications. The mobile phases were ultrapure water acidified to pH 2.10 with phosphoric acid (A) and methanol: acetonitrile (90:10, v/v) mixture (B). The flow rate was 1.0 mL/min and the total run time was 73 min. The elution gradient was as follows: 95% A and 5% B for 2 min, 75% A and 25% B in 8 min, 60% A and 40% B in 10 min, 50% A and 50% B in 16 min, 0% A and 100% B in 14 min. This last composition was sustained for 10 min and then returned to the initial composition within 13 min. The quantification PC was performed with calibration curve for each available commercial standard. The phenolic alcohols (hydroxytyrosol, tyrosol), oleuropein, phenolic acids (*p*-qumaric, *t*-ferrulic, caffeic and vanilic acids), catechin and pinoresinol were detected at 280 nm, while apigenin and luteolin were detected at 335 nm. Results were expressed as $mg.kg^{-1}$.

*Volatile compounds (VC) analysis*

A 2 g of oil sample was weighed in a 20 mL SPME vial (Supelco) and 10 µL of isobutyl acetate (500 $mg.L^{-1}$) solution was added as an internal standard (IS). The vial was closed with a screw cap with a silicone septum and placed into the tray of the autosampler. The rest of SPME operations were applied on the autosampler. The sample vial was maintained in the heating block at 40 C° for 30 min under an agitation at 220 rpm. Then, a divinlybenzene/carboxen/polydimethylsiloxane (DVB/CAR/PDMS) fiber (2 cm, 50/30 µm, Supelco) was inserted to the vial and exposed to the headspace of the vial at the same conditions for 25 min.

After extraction, the VC were desorbed thermally from the fiber in the injection port of the GC at 250 C° for 5 min, with a splitless mode. The separation was achieved with a DB-HeavyWax column (0.25 µm, 60 m x 0.25 mm) (Agilent Technologies). Hydrogen was used as the carrier gas at a flow rate 1.05 ml.min-1. The oven temperature was first kept at 40 C° for 2 min, then increased to 80 C° at a rate of 3 C°.min-1 and held at this temperature for 1 min. and then was programmed to 240 C ° at a rate of 5 C°.min-1 and held at this temperature for 6 min. The temperatures of ion source and the interface of MS were 201 C° and 250 C°, respectively. Electron ionizing (EI mode) energy was recorded at at 70 eV. Mass scanning range was m/z 20-450.

The identification of VC was performed by comparing their mass spectra with those of NIST11 and Wiley9 mass spectral libraries and literature. Additionally, the many identifications were confirmed by comparing VC retention indices (Kovats Index) with those available in the literature. The quantifications of VC were calculated by matching the peak area of each compound to the calibration curves of IS, and expressed as mg per kg of sample.
